# Using ex vivo arterial injection and dissection to assess white matter vascularization

**DOI:** 10.1038/s41598-022-26227-6

**Published:** 2023-01-16

**Authors:** Mykyta Smirnov, Igor Lima Maldonado, Christophe Destrieux

**Affiliations:** 1 UMR 1253, iBrain, Université de Tours, Inserm, Tours, France; 2 CHRU de Tours, Tours, France

**Keywords:** Brain, Neural circuits, Anatomy, Nervous system

## Abstract

Advances in the techniques for assessing human cerebral white matter have recently contributed to greater attention to structural connectivity. Yet, little is known about the vascularization of most white matter fasciculi and the fascicular composition of the vascular territories. This paper presents an original method to label the arterial supply of macroscopic white matter fasciculi based on a standardized protocol for post-mortem injection of colored material into main cerebral arteries combined with a novel fiber dissection technique. Twelve whole human cerebral hemispheres obtained *post-mortem* were included. A detailed description of every step, from obtaining the specimen to image acquisition of its dissection, is provided. Injection and dissection were reproducible and manageable without any sophisticated equipment. They successfully showed the arterial supply of the dissected fasciculi. In addition, we discuss the challenges we faced and overcame during the development of the presented method, highlight its originality. Henceforth, this innovative method serves as a tool to provide a precise anatomical description of the vascularization of the main white matter tracts.

## Introduction

Advances in the techniques for assessing cerebral white matter have contributed to greater attention devoted to structural connectivity in recent years^1–7^ and its importance for understanding the pathophysiology of brain diseases^8^. This essential anatomical substratum completes the interpretation of normal and pathological functional data obtained from imaging or neuropsychological assessment. As important as a brain area may be for accomplishing a given task, it can only participate in that task if it is connected to other cerebral regions. In this context, research efforts on the anatomy of the white matter fasciculi have considerably gained momentum over the last decade.

Another important element to promote the transfer of research results to clinical practice is integrating information on vascular anatomy. Stroke is the main mechanism of damage to these structures, but vascularization is not incorporated into most connectome studies. In vascular neurology, classical studies^9,10^ on stroke severity have highlighted the importance of volume of infarction or location of occlusion in relation to the cortical localizationist model. These approaches are reductionist since they minimize the importance of the (often predominant) infarcted white matter and diverge from the notion of functional networks. A better knowledge of the relationships between white matter fasciculi and vascular territories seems essential for advancing our understanding of the consequences of cerebral ischemia and promoting the transfer of research results on connectivity to clinical practice in vascular neurology.

In the first half of the XXth century, Ludwig and Klingler^11^ implemented a method to demonstrate fiber bundles in the *post-mortem* brain based on a freezing–thawing cycle of formalin-fixed cerebral hemispheres. This procedure greatly facilitated the dissection of fasciculi by creating gaps among white matter fibers while preserving their overall architecture^12^. The technique has become very popular both as a research tool and a pathway for learning microsurgical anatomy. Numerous variants^1,6,7^ have been developed in the last 20 years, but, again, vascular structures have not been approached in any of them. On the contrary, to the best of our knowledge, the totality of fiber dissection protocols in human brains includes a step of removal of leptomeninges and vessels. Insofar as the exposure of fibers is obtained completely unrelated to the study of cerebral vascular territories, a vital opportunity to cross-fertilize research efforts is wasted.

Ultimately, little is known about the vascularization of most white matter fasciculi and the fascicular composition of the vascular territories^8^. The boundaries and variants of the deep vascular territories are much lesser known than the cortical territories. Until recently, detailed descriptions of the vascular territories in the white matter have been available only for a few areas, the other being often delineated by arbitrary (often straight) putative boundaries deduced by extending the cortical territories up to the ventricular walls^8^. The procedures of ex vivo vascular injection used in classical anatomical works have often been described with a low level of detail and non-standardized protocols, which may explain part of the variability of results^13^.

This paper presents an original method to label the arterial supply of macroscopic white matter fasciculi based on a standardized protocol for *post-mortem* injection of colored material combined with a novel fiber dissection technique.

## Materials and methods

Twelve human cerebral hemispheres (6 left, 6 right) were obtained from 7 subjects (2 female, 5 male, mean age: 88.4 ± 10.7 years, min.: 66, max.: 98) enrolled in our institution’s body donation program. Written informed consent was obtained from the participants to the use of their bodies for educational or research purposes in which the anatomy laboratory is involved. This study was approved by the board of the University of Tours Body Donation Program and performed in accordance with the relevant guidelines and local regulations.

### Specimen preparation

#### Brain extraction

We extracted the brains within 48 h *post mortem* to limit tissue deterioration. The internal carotid arteries (ICAs) and basilar artery (BA) were sectioned as distal as possible from the brain during the extraction. The dura mater was left in place due to its tight adherence to the calvarium in the elderly. Immediately after extraction, the brains were immersed in cold water (+ 4 °C) to keep their shape and preserve unfixed fragile cerebral tissues. To limit deformation of the specimens induced by contact to the water tank, we attached a thread coursing between the BA and the pons to the superior edges of opposite walls of the container. The thread’s tension was controlled to ensure complete immersion of the specimen without contact with the container walls.

#### Vessel catheterization and brain prefixation

We used silicone catheters (outer diameter 1–1.5 mm) and soft vinyl baby bird feeding tubes (outer diameter 2–3 mm) for selective catheterization of the main cerebral arteries. Catheters fitting the vessel size (usually 1.5 mm for the anterior cerebral arteries and 3 mm for the middle cerebral arteries and BA) were equipped with 3-way valves. They were flushed with water, and valves were closed to ensure no residual air was present in their lumen. Then, arteries were catheterized while immersed. Vessels were ligated around the inserted catheters. The catheter-vessel interface was then dried with a cotton pad and secured with cyanoacrylate glue (Super glue-3 power gel, Loctite, Henkel France Adhesives, Boulogne-Billancourt, France).

**Figure 1:**
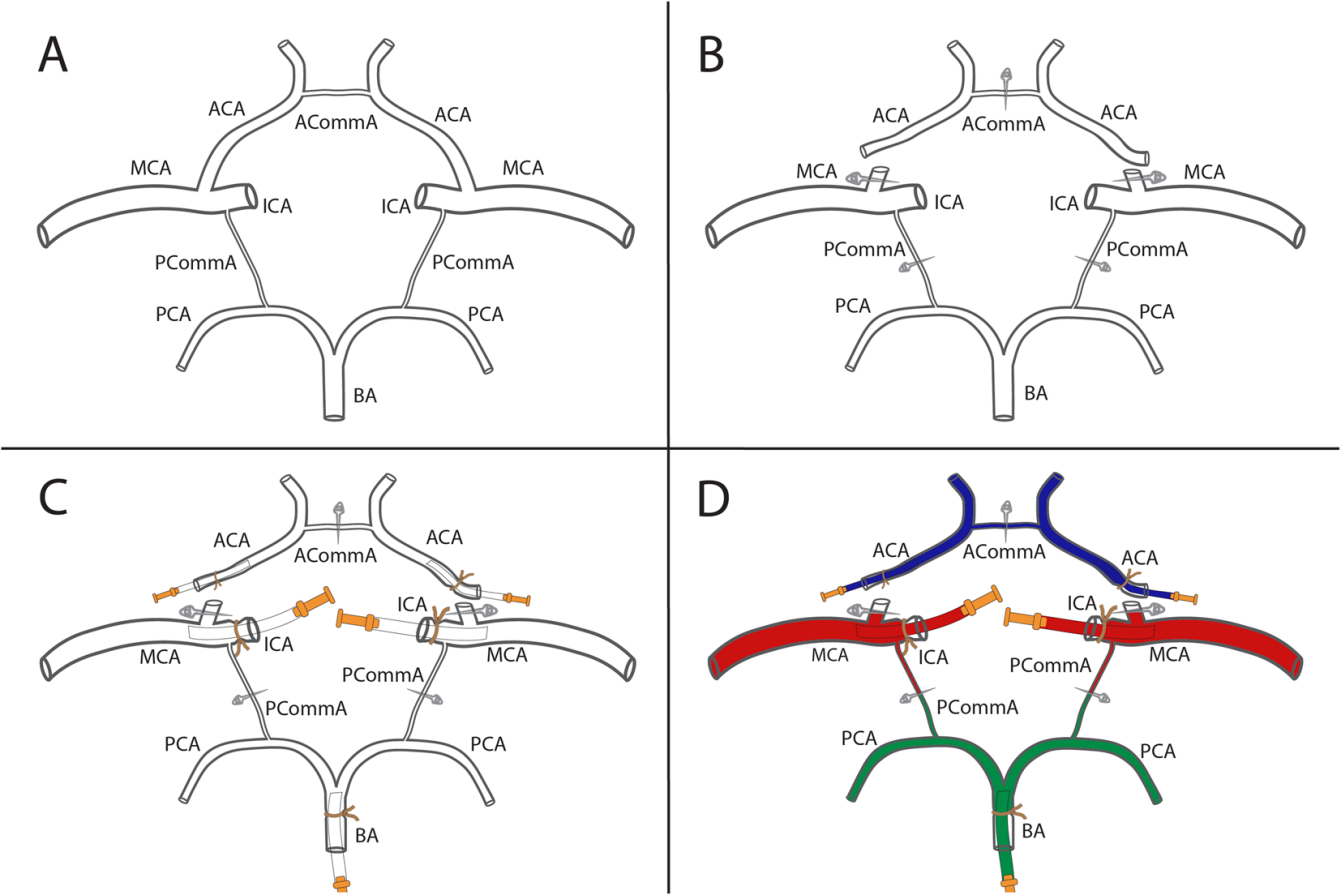
Catheterization of the cerebral arterial circle. (**A**): Cerebral arterial circle before catheterization. (**B**): The anterior and posterior communicating arteries were clipped. The anterior cerebral arteries were proximally sectioned. (**C**): Catheters were inserted and ligated in the corresponding arteries. (**D**): The cerebral arterial circle after arteries were flushed with isotonic saline and injected with colored gelatin solutions. We used blue for injection of the anterior, red for the middle, and green for the posterior cerebral arteries. *ACA* anterior cerebral artery, *ACommA* anterior communicating artery, *BA* basilar artery, *ICA* internal carotid artery, *MCA* middle cerebral artery, *PCA* posterior cerebral artery, *PCommA* posterior communicating artery.

The catheterization procedure was as follows (Fig. 1):

- (1) *Anterior cerebral arteries (ACAs)*. Using a surgical aneurysm clip, we occluded the precommunicating (or A1) segments of the ACAs at their origin from the ICAs. A1s were then sectioned distally to the clip, and catheters were inserted into the arteries, not occluding perforating arteries. The anterior communicating artery (ACommA) was clipped to prevent medium exchanges between ACAs territories during the injection.
- (2) *Middle cerebral arteries (MCAs)*. A catheter was inserted within each supraclinoid ICA and advanced just distal to its division into the MCA and ACA, at the proximal origin of the sphenoidal (or M1) MCA segment. Catheters were introduced to preserve flow within perforating branches located more distal. The posterior communicating arteries (PCommA) were clipped to prevent medium exchanges between MCA and PCA territories.
- (3) *Posterior cerebral arteries (PCAs)*. The BA was sectioned at the pontine level and catheterized up to close to its bifurcation.

To wash residual blood and check the quality of catheterization (absence of leaks and occlusions), we successively flushed each of the catheterized arteries with 200 ml of isotonic saline using a 50 ml syringe. We opened the 3-way valves only for a short time to let the flow during the injection. Between injections, they remained closed to keep the inner pressure in each arterial tree.

Next, we injected 150 ml of 4% formaldehyde aqueous solution (Formaldehyde 4% w/v buffered at pH 6.9 RS, Carlo Erba Reagents, Cornaredo, Italy) per hemisphere to obtain primary vessel fixation and enhance the subsequent immersion fixation of the brain^14^.

#### Injection material preparation

To visually distinguish vascular territories, arteries were injected with a gelatin aqueous solution colored with dry pigments: red (SP rouge 55, azo pigment, Color Rare, Villenave d’Ornon, France), blue (SP bleu 7, copper-phthalocyanine pigment, Color Rare, Villenave d’Ornon, France), and green (SP vert 8, halogenated copper-phthalocyanine pigment, Color Rare, Villenave d’Ornon, France). We used blue color for the injection of the ACAs, red for the MCAs, and green for the PCAs.

Preparation of injection material consisted of two steps:

1. Dyes were diluted in distilled water (pigment concentrations were 0.5% for blue and red, 0.25% for green). Then, we heated the mixture using a magnetic hotplate stirrer (IKA RH basic 2, Staufen, Germany) to 32 °C and let it cool down to ambient temperature.
2. Gelatin from porcine skin (gel strength 300 Bloom, Type A, Sigma-Aldrich, St. Louis, MI, USA) was added to obtain a 5% gelatin concentration. We stirred the mixture at the ambient temperature to get a visually uniform distribution and heated it again to 50 °C while stirring. Once the mix was ready, it was transferred to the injection bottles (gas washing Drechsel bottles).

#### Injection

**Figure 2:**
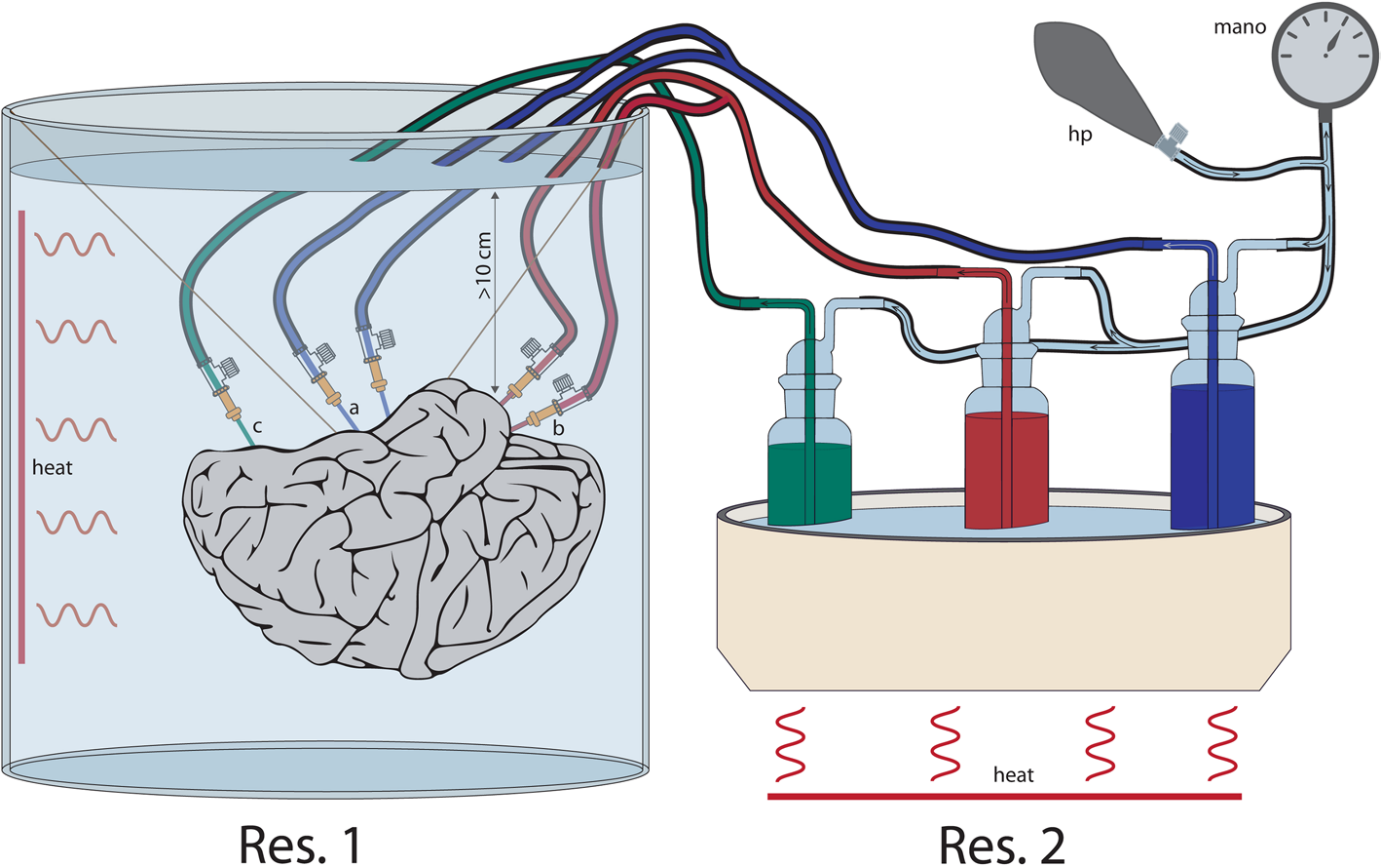
Schematic representation of a simultaneous injection apparatus. It consisted of two reservoirs. The bigger (**Res. 1**) was a cylindrical glass aquarium accommodating the floating brain. The smaller (**Res. 2**) was a paraffin mounting bath containing glass gas washing bottles. In both reservoirs, the water temperature was 40 °C to prevent the gelatin solution from early solidification. To maintain the desired water temperature in the left tank, we used a low-temperature cooking machine with a built-in thermostat which simultaneously agitated/heated the water; the smaller reservoir had a built-in heating element and thermostat. The gas washing bottles were connected with silicon tubes to the catheters on one side (the anterior (a) and middle (b) cerebral arteries through the Y-connectors, the basilar artery (c)—directly) and a hand pump with a manometer on the other.

To reproduce physiological conditions during injection (blood pressure and competition of blood flow among adjoining arterial territories), we adapted to gelatin the method initially proposed by van der Zwan and Hillen for plastic^13^. The injection apparatus (Fig. 2) consisted of two reservoirs filled with warm 40 °C water to prevent gelatin from early solidification:

- The bigger one, a cylindrical aquarium, contained the brain submerged under 15 cm of water to simulate “venous pressure^13^”. Water was simultaneously agitated and heated by a device including a built-in heating system, a thermostat, and a propeller (Immersion sous vide precision cooker, ANOVA, San Francisco, CA, USA).
- The smaller reservoir, a paraffin section mounting bath (Electrothermal, Staffordshire, UK) with a built-in heating element and thermostat, contained gas washing Drechsel bottles made from glass and filled with colored gelatin. A short inlet and a long outlet tube passed through the bottle cap. The outlet tube extended almost to the bottom of the bottle, so its lower end was immersed in liquid gelatin.

Soft silicone tubes were mounted onto arterial catheters on one end and connected to the outlet tubes of the corresponding bottles on the other (both the ACAs and MCAs through the Y-connectors). Finally, a single hand pump and analog manometer were attached to inlet tubes. Before injection, the air was evacuated from the system to avoid flow stops.

Injection of colored gelatin began at low pressure (20 mmHg) to stabilize the whole circuit. Then, the pressure was increased to 140–160 mmHg to create a continuous flow of injection material towards the brain. The injection was continued till visually assessed filling of pial arteries was satisfactory and ended by closing the catheters’ 3-way valves.

The injected brains were then immersed in cold water (+ 5 °C) for 10 min to speed up the gelatin setting and, subsequently, in a 4% buffered formaldehyde solution for 2 months at ambient temperature. As described, the brains were suspended by a thread around the basilar artery to limit distortion induced by contact with the container walls.

#### Preparation for fiber dissection

After 2 weeks of immersion fixation, the brains became less fragile, allowing manipulation. We removed clips and remaining parts of catheters and placed surgical gauzes within the longitudinal fissure and between the cerebellum and occipital lobes. The gauzes favored fixation by promoting formaldehyde diffusion in areas where hemispheres, under normal conditions, were in close contact together or to the cerebellum. At the same time, the fixation solution was renewed.

Two months after injection, brains were placed in a freezer (− 23 °C) for 1 week, then in a refrigerator (+ 5 °C) for slow thawing for 3–5 days. After that, the brainstem and cerebellum were removed from the specimen, and hemispheres were separated. Each hemisphere with its medial surface downwards was placed on a 3D-printed polylactic acid (PLA) holder. Then, we filled the PLA holders with a 2 cm layer of melted black-dyed paraffin (Fig. 4C). Finally, the specimens fixed onto the holder by the solidified paraffin were immersed into a renewed 4% formaldehyde bath.

### Fiber dissection

**Figure 3:**
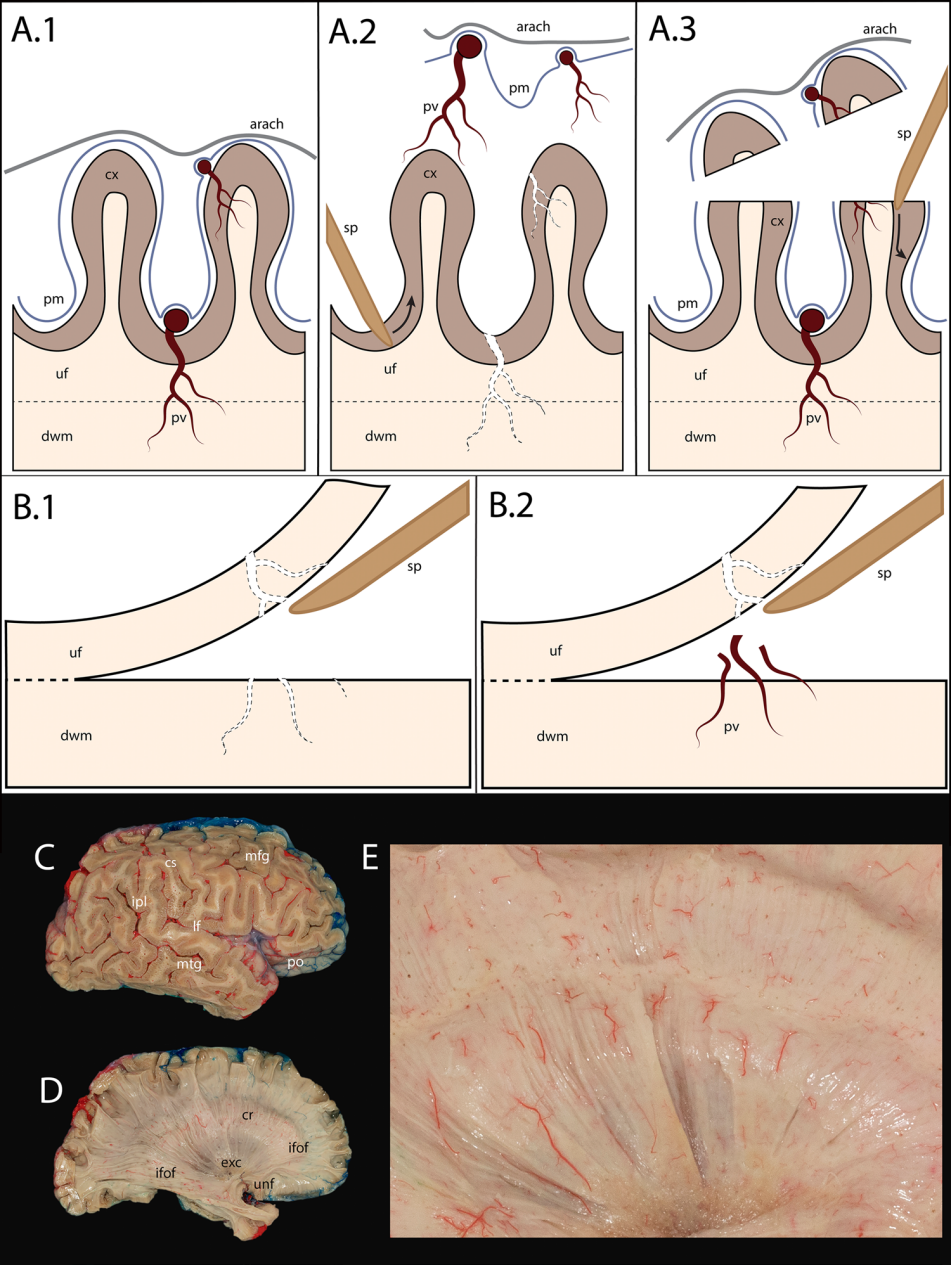
Technique of fiber dissection with preservation of the white matter vasculature. (**A.1**): Parenchyma and vessels before dissection. The pia mater covers both the cortex and subpial vessels, to which it is firmly attached. (**A.2**): Removal of the leptomeninges for classical dissection results in vessel avulsion, which hinders the study of white matter vasculature. (**A.3**): Sectioning of the superficial-most millimeters of gyri enables vessel preservation. (**B.1**): Classical fiber dissection with no vessels left. (**B.2**): Fibravasc technique. Vessels slip through the white matter layers removed and keep to be anchored to the deeper ones. If needed, vessels can be easily divided in-between the two layers. (**C**): Orthogonal view of the dissection step 1 (corresponds to the illustration (**A.3**)). (**D**): Orthogonal view of the dissection step 8 (corresponds to the (**B.2**)). (**E**): Cropped and enlarged picture contains the corona radiata, external capsule, and claustrum (from top to bottom) and presents vessels protruding from the white matter, as shown in (**B.2**). In **(C**,**D** and **E**), preserved vessels expose arterial distribution territories (blue—the anterior, red—medial, green—posterior cerebral artery). *arach* arachnoid mater, *cl* claustrum, *cr* corona radiate, *cs* central sulcus, *cx* cortex, *dwm* deep white matter, *exc* external capsule, *ifof* inferior fronto-occipital fasciculus, *ilf* inferior longitudinal fasciculus, *ipl* inferior parietal lobule, *lf* lateral fissure, *mfg* medial frontal gyrus, *mtg* medial temporal gyrus, *pm* pia mater, *po* pars orbitalis, *pv* pial vessel, *sp* spatula, *uf* U-fibers, *unf* uncinate fasciculus.

According to a standardized protocol, specimens fixed to PLA holders underwent modified fiber dissection to expose white matter fibers and the injected vasculature. In the original Klingler's method, leptomeninges and vessels (Fig. 3A.1) are removed just before^1^ or after^15^ freezing, but in any case before dissection (Fig. 3A.2). Since we had to preserve vessels as much as possible, dissection began by a careful examination and gentle sectioning of the outer 3–5 mm layer of cerebral gyri, containing the cortex and leptomeninges (Fig. 3A.3). This step preserved the sulci, the cortex within the sulci, and the sulcal vessels (Fig. 3C). The remaining cortex, made spongy and brittle by the freezing–thawing process, was then removed under optical magnification.

Next, we removed the U-fibers and proceeded with progressive exposal of white matter tracts (Fig. 3B.2), always preserving the vasculature marked by the colored medium (Fig. 3E). For this purpose, sectioning of small arteries was sometimes necessary as we advanced to the depth to avoid inadvertent pulling and losing part of the marking (Fig. 3B.1). The following fiber bundles were studied: the superior longitudinal fasciculus (SLF, specifically SLF-II and SLF-III), arcuate fasciculus (AF), inferior longitudinal fasciculus (ILF), inferior fronto-occipital fasciculus (IFOF), uncinate fasciculus (UF), cingulate fasciculus, optic radiations, thalamic radiations, anterior commissure, and callosal radiations.

**Figure 4:**
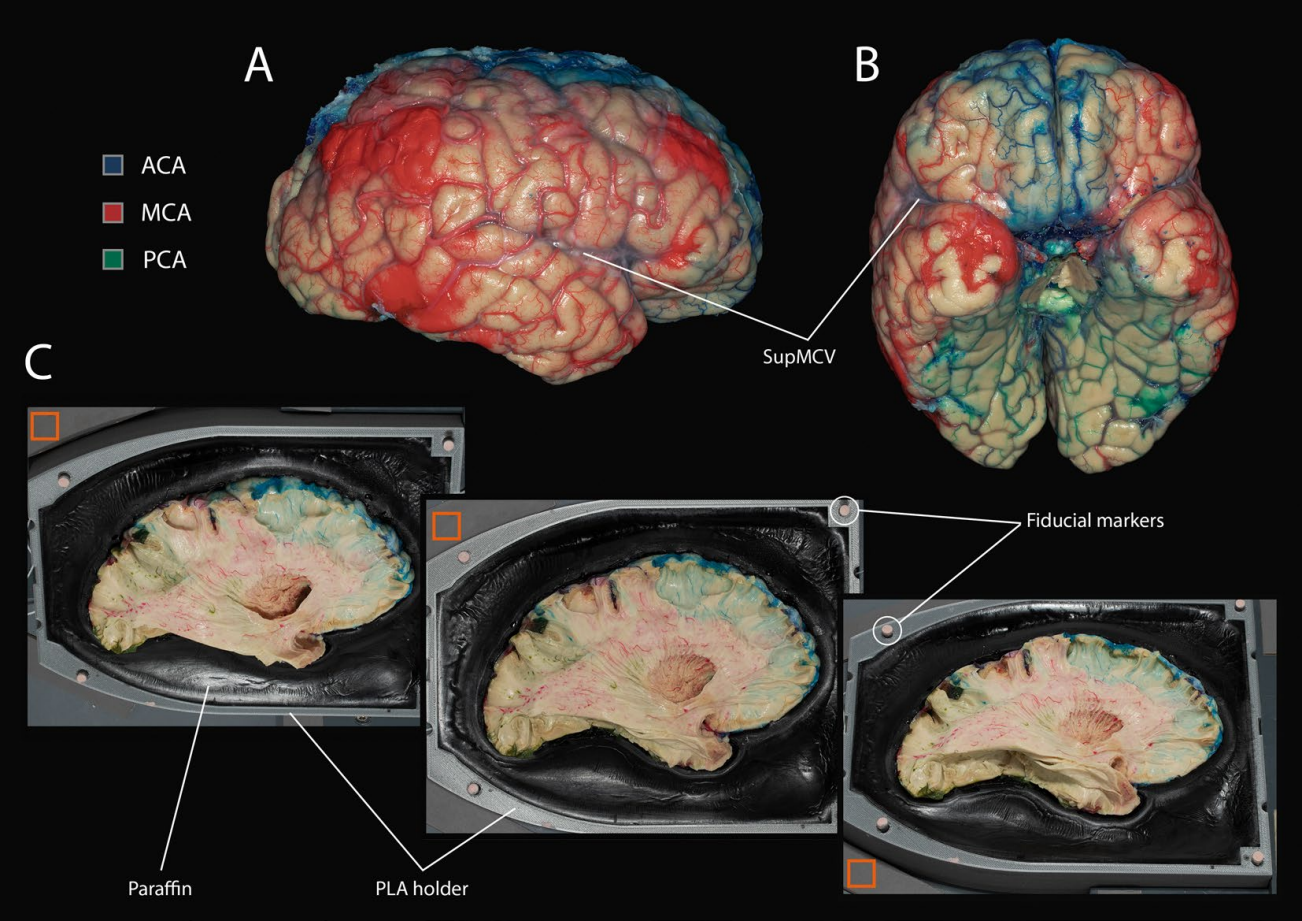
Injection results and 3D-printed holder. (**A**): Right lateral and (**B**): Anterior-inferior views of a human brain injected with colored gelatin (ACAs—blue, MCAs—red, PCAs—green). Pial arteries were filled with the pattern expected from the literature, while the superficial SupMCV remained collapsed. (**C**): Three pictures were taken from different angles to maximize the captured surface: oblique superior (left), orthogonal (middle), and oblique inferior (right). Light gray 3D-printed polylactic holder was filled with black paraffin to fix the position of the specimen. Although the relative position of the holder and cameras was maintained constant by a rigid support, additional fiducial markers (light pink circles) further improved the alignment of different steps images. Reference gray (RGB: 113, 113, 113) color patches (orange squares) are present to correct white balance and exposure precisely. *ACA* anterior cerebral artery, *MCA* medial cerebral artery, *PCA* posterior cerebral artery, *SupMCV* superficial middle cerebral vein.

Dissection was periodically interrupted to trace each significant step of the process (Fig. 3C, Fig. 3D). We used two digital single-lens reflex cameras (Canon 80D, Canon, Tokyo, Japan), each equipped with a macro lens (Canon EF-S 60 mm f/2.8 Macro USM, Canon, Tokyo, Japan) and connected to a single ring flash (Canon Macro Ring Lite MR-14EX II, Canon, Tokyo, Japan). These cameras were fixed above the rotating PLA holder support: the first one with a flash was orthogonal to the specimen, the second—oblique. We acquired three photographs from different viewpoints for each dissection step to maximize the visible specimen surface: orthogonal, oblique superior, and oblique inferior. Oblique views were changed by rotating the PLA holder support by 90° (Fig. 4C). The cameras were controlled by Capture One 12 software^16^ with the following parameters: shutter speed—1/160, aperture—16, ISO speed—200, white balance—manual, color temperature—5500, drive mode—single frame, AF mode—manual, metering mode—evaluative. Pictures were taken with the laboratory lights off. Each image included a gray patch (RGB values: 113, 113, 113) used as a reference to correct white balance and exposure and four fiducial markers used to check the proper alignment of the photographs across dissection steps. The main white matter fasciculi and arterial distribution territories were then studied.

## Results

### Injection

No segment of the cerebral arterial circle was missing in the brains included in this study. Only the size of the arteries varied, especially of the ACAs, but was consistent on both sides of a given brain. A continuous injection pressure of 140–160 mmHg provided satisfactory injections, i.e., the whole brain surface was injected (Fig. 4A,B), although a slight inter-individual variation was observed.

Several arguments are in favor of the colored injection material stability: (1) when immersed in cold water, gelatin setting occurred instantly, thus preventing possible post-injection leaking; (2) the first 4% formaldehyde bath was slightly colored, whereas the subsequent baths remained colorless; (3) similarly, we didn’t observe any significant change in the color/density of the injected vessels at the brain surface between the different baths renewals. This pleads for an initial washing of excess pigments from the brain surface without any secondary diffusion from the injected vessels.

### Fiber dissection

Combination of rigid support, PLA holder, paraffin (Fig. 4C), and handling the specimen with great care provided its consistent position from one photograph acquisition to another, as demonstrated by fiducial markers alignment across dissection steps. From 13 to 15 steps of dissection were photographed per hemisphere, depending on its size and dissection complexity.

Notably, gelatin injection of specimens had no significant impact on Klingler’s dissection process: we did not observe any change in the dissection feasibility for short or long white matter fibers after such a procedure. The only requirement was to be careful not to pull out vessels from the white matter while removing the leptomeninges and superficial cortex. Projection fibers were intentionally neglected during the dissection of the main white matter association bundles to keep the latter intact.

A detailed description of the morphology of all dissected white matter fiber tracts is out of the scope of this methodological paper. We provide a brief overview of their overall organization, consistent with previous descriptions^6,7,11,15,17–19^.

Following the dissection protocol described by Zemmoura et al.^1^ we exposed superficial (short association U-fibers) and middle groups of the cerebral white matter fiber system^15^. The latter consisted of the (1) SLF-II and SLF-III, respectfully, connecting the middle and inferior frontal gyri with the inferior parietal lobule, (2) AF in a C-shape appearance, connecting the inferior frontal gyrus to the temporal cortex, (3) ILF, underlying the inferior temporal and fusiform gyri and connecting the temporal and occipital poles, (4) IFOF in an hourglass-shaped appearance, connecting the cortex of the frontal and occipital lobes, (5) UF, linking the orbitofrontal cortex and the cortex of the temporal pole, and (6) sagittal stratum.

**Figure 5:**
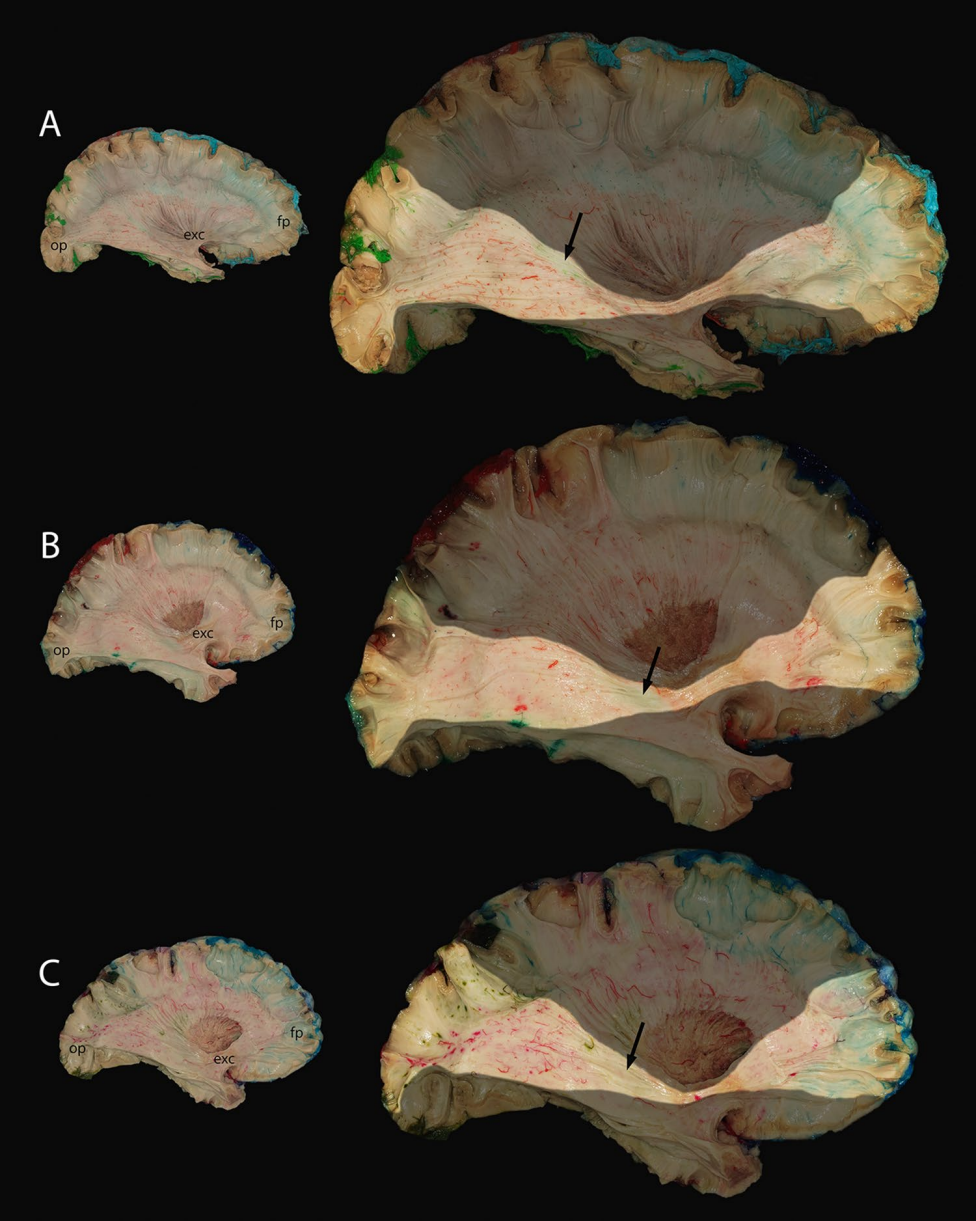
Example of the application of the Fibravasc protocol to study the arterial supply of a specific white matter structure: the inferior fronto-occipital fasciculus (IFOF). Overview (left column) and enlarged contrasted views (right column, structures outside the area of interest were shaded) of three out of twelve specimens. The territory of the ACA is blue, MCA—red, PCA—green. The frontal segment of the IFOF, anterior to its trunk, was supplied by the MCA and ACA in its posterosuperior and anteroinferior portions, respectively. The relative contribution of the ACA and MCA was susceptible to significant interindividual variability (for example, considerably greater contribution of the ACA in (**A**)). The IFOF’s trunk was located in the ventral part of the external and extreme capsules, just dorsal to the limen insulae. It was vascularized by the MCA in all specimens. The temporo-occipital segment, which extended from the IFOF's trunk to its posterior endings, was supplied by the MCA and PCA. Branches of the latter contributed to a variable extent to the vascularization of the occipital and parietal posterior endings ((**B**) shows the smallest territory). For some specimens, we observed a circumscribed area supplied by the PCA (arrowhead) in the anterior portion of this segment (in (**C**)—the largest, oval-shaped area). *ACA* anterior cerebral artery, *exc* external capsule, *fp* frontal pole, *MCA* middle cerebral artery, *op* occipital pole, *PCA* posterior cerebral artery.

Next, the putamen and then globus pallidus were removed, thus, exposing the internal capsule and the anterior commissure. We proceeded the dissection further until the structures of the vertical and central white matter fiber system groups^15^ were taken out, finally leaving only the cingulum and medial superficial U-fibers. It was possible to follow white matter vascularization and observe the distribution territories of major cerebral arteries during every dissection step. As an illustration, the IFOF vascularization is described in Fig. 5.

## Discussion

Although vascularization of the white matter is paramount in understanding clinical symptoms after a stroke, traumatic brain injury, or postoperative ischemia, it was relatively neglected in the literature. While the information on the arterial distribution of the internal capsule (mostly projection fibers) can be found^20,21^, the territories of many regions, such as the centrum semiovale and sagittal stratum, or more generally speaking of the long association tracts are not clearly delineated or only partially described^8,20^. In this paper, we propose a novel method to simultaneously dissect major white matter fibers tracts and directly assess their arterial distribution from injected anatomical specimens. We deliberately chose to inject only main cerebral trunks (the ACAs, MCAs, and PCAs) without injecting, for instance, the anterior choroidal artery, whose territory was extensively studied.

Several attempts were made to study the white matter vascularization using various methods, injection materials, and dyes. Heubner^22^, Duret^23^, and Charcot^24^ were pioneers in successively injecting gelatin, i.e., one by one, into major cerebral arteries but did not fully describe the protocol they used for specimen preparation. To inject small-caliber arteries (the anterior choroidal artery, posterior communicating artery perforators), Kolisko^25^ changed the gelatin to oily substances (Teichmann’s cold mass and Kadyi’s olive oil mass) colored with compatible dyes (carmine, finest chrome yellow, and Berlin blue).

At the beginning of the twentieth century, Beevor realized that successive injections were too far from physiological conditions to display the “real” arterial distribution territory; injecting a single vessel creates a pressure gradient directed towards other vascular trees, thus opening leptomeningeal anastomoses and extending injected territory artificially. He then developed a simultaneous injection technique of colored gelatin^26,27^ and tried various pigments; the ones with the most satisfactory properties were carmine, acridine yellow, and naphthol green.

This simultaneous method was nevertheless not broadly adopted. For instance, in 1924, Shellshear^28^ implemented Beevor’s^26^ gelatin preparation recipe in his studies, but not the injection technique, as he used syringes for consecutive injections instead of Beevor’s pressure apparatus.

In the second half of the twentieth century, researchers used more contemporary methods, such as ex vivo radioangiography^29–35^ and scanning electron microscopy^34–37^, and new injection materials, such as low viscosity resin^34–36^, silicone^37^, latex^40^, and plastic^13^. The latter, specifically Araldite F, was used by van der Zwan and Hillen^21^ whose methodology^13^ was the first standardized and entirely described in the literature. The authors stated that to obtain reliable results, the procedure of preparing the brain, injecting the arteries, the post-injection procedures, and data collecting should be conducted under optimal and, importantly, standardized conditions^13^. We adapted their method (simultaneous injection, controlled pressure) to be used with gelatin, a dissection-compatible material.

Gelatin is affordable, environment-friendly, and easy to operate when complying with proper temperature conditions. It can be colored with various water-soluble dyes. Due to its organic nature and softness, gelatin can be formalin-fixed alongside the brain and is suitable for subsequent dissection. At 40 °C, it is fluid enough to flow through and fill the vessels. But, the price to pay for this fluidity is leaking during injection, especially on the venous side: since draining veins of the extracted brain are no longer connected to venous sinuses, they directly drain to the injection tank’s water. Moreover, since the extracted brain is no longer submitted to intracranial pressure, the injection medium flows along the path of least resistance, resulting in the poor injection of deep parenchymal vessels. One option would be not to extract the brain before injection, prohibiting any selective arterial injections and visual inspection of the injection quality. To overcome this challenge, we immersed the brain under 15 cm of water to simulate physiological intracranial pressure, as van der Zwan and Hillen proposed^21^. This manipulation limited leaks through widely open veins in proximal territories and enhanced filling of deep and distant arterial terminals.

At ambient temperature, gelatin sets too soon and blocks the flow, limiting the injected territory. We added heating elements to the injection apparatus’s water reservoirs to prevent early gelation. They maintained the gas washing bottles and brain at desired temperature all the injection procedure long. In addition, we shortened silicone tubes to minimize the time gelatin spent outside the conditioned environment. After injection, specimens were rapidly cooled to accelerate the gelatin setting and limit tissue deterioration, which high temperature may facilitate^13^.

Other injection materials^13,34–42^ that do not require heating of a specimen were proposed, but each of them faced the limitation if used with white matter dissection. After polymerization, injected vessels filled with silicon and latex act as “rubber bands”. This mechanical property makes them more resistant to manipulation and more suitable for superficial descriptive anatomy. Still, it conversely limits deep specimen dissection and negatively impacts section cutting of paraffin-impregnated blocks. Plastic materials and resins are rigid and can be mainly used to prepare vessel casts for scanning electron microscopy.

Dyes used with gelatin had to comply with several criteria: (1) good solubility in water, (2) miscible with gelatin, (3) mild heating resistance, (4) no change in color during and after formalin-fixation and good tinting strength, (5) absence of diffusion in perivascular tissues and surrounding liquids after the gelatin sets, (6) absence of toxicity, (7) availability on the market, (8) and, if possible, non-ferrous components allowing ex vivo MRI investigation. We tried various liquid acrylic inks, but, unfortunately, none of them met all the aforementioned criteria. This way, SP line dry pigments by Colorare, described in the materials and methods section, showed the best performance by full compliance.

During the brain injection, constant pressure was kept in the injection apparatus system to comply with a contemporary model of cerebral autoregulation^43^. Together with the simultaneous injection, this manipulation simulated the physiological conditions to a greater extent.

Cortical arterial distribution territories observed in the present study were variable but expected. By contrast, deep white matter territories were more complex and unpredictable than considered in the literature^8^, not necessarily following and mirroring the cortical distribution. Considering the different pressure levels, in a preliminary stage of method development, lower injection pressures (80–100 mmHg and 120–140 mmHg) did not enable injection material to reach the majority of small vessels even while no obvious clots were observed. Pressure higher than 160 mmHg was considered excessive, as it could lead to vessel damage resulting in “gelatinorrhagia” into cerebral tissues and a malfunction of the injection apparatus. Since this paper focuses on the complex methodology and presents it in detail to ensure its reproducibility, we only provided a few examples of the IFOF vascularization patterns (Fig. 5). The comprehensive mapping of arterial distribution territories of a given fasciculus (or group of fasciculi) is intended for a forthcoming publication.

Our method permitted the dissection of main white matter association tracts without significant changes of the dissection process, except at the first steps. Classical Klingler’s dissection^44^ commences with removing the leptomeninges with vessels and subsequent peeling of the entire cortical gray matter in the area of interest before the actual white matter dissection. The pial arterial network sends numerous branches to the underlying white matter^8^ that are teared-off from the parenchyma alongside the leptomeninges in this initial step. We, first, gently resected only the outer 3–5 mm layer of the cerebral gyri underlying leptomeninges to preserve vessels. This manipulation allowed the sulco-gyral pattern to be retained and the remaining cortex (and leptomeninges) removed “from the inside” while bluntly breaking or cutting the vessels on the border with the white matter instead of pulling them out.

The role of the white matter in the pathogenesis of neurological deficits is nowadays unquestionable. Many symptoms greatly depend on the direct injury of the white matter fasciculi or are the consequence of dysfunctions of the cortical areas they normally connect. For instance, even a small lesion within the extreme and external capsule, accommodating the IFOF and UF, disrupts the ventral semantic pathway circuits. Such IFOF lesions are devastating for the patient’s daily life since they induce verbal semantic deficit in the dominant and non-verbal semantic deficit in the non-dominant hemisphere^45^. Symptoms observed in Lelong’s and Leborgne’s brains described by Broca in his seminal work^46^ were for long related to lesions limited to the left frontal cortex. Their MR examination nevertheless showed wider lesions, including a large perisylvian white matter region^47^. Thus, the observed symptoms were secondary to significant disturbances in parallel and, possibly, overlapping neural circuits involving the gray and also—perhaps mainly—white matter. An essential consequence of this paradigm switch is that parts of the brain, usually considered critical in the classical localizationist theory, can be surgically resected when the involved networks ensure compensation^46^.

Consequently, interest in white matter fiber tracts dramatically grew in the last decades and benefited from methodological developments giving access to their anatomy *in* and ex vivo. From Klingler’s seminal papers^11,44^, dozens of dissection papers were published^1,6,48,49^, and the tissue modifications induced by the freezing–thawing process were elucidated^12^. More recently, diffusion-weighted imaging became an effective tool for exploring the white matter anatomy *in*^17^ and *ex vivo*^50,51^, at a spatial resolution as small as 0.1 mm, and to access white matter microstructure and connectivity^52,53^. Others methods, based on the optical properties of the white matter, such as polarized light imaging^54,55^ and optical coherence tomography^56,57^, provide information on its anatomy at the micrometer scale. As previously mentioned, gelatin is a versatile medium having mechanical and magnetic properties that permit its use with various methods, from dissection to section cutting. A combination of ultra-high field ex vivo MRI would, for instance, provide exquisite information on the white matter microstructure and vascular territories.

Modern neuroimaging aims to predict tissue consequences (and infer clinical prognosis) after a stroke. However, most clinical studies on stroke rely on the volume of the infarcted tissue or its cortical relationships (theories of cortical localization)^58^. As a result, these approaches minimize the—often predominant—role of the white matter towards the cortex^47^. Thus, the modern anatomy of the main white matter bundles remains apart from everyday vascular neurology and is not used to predict the long-term stroke clinical course. Therefore, neurologists’ better knowledge of the relationships between bundles and vascular territories appears essential to improve understanding of stroke symptomatology and prognostic evaluation. In addition, defining the injured white matter tracts after a stroke will improve patient stratification and care personalization.

One of the possible limitations of this study is the age of the participants (mean: 88.4 years) which is the consequence of how we obtained brains (body donation). Death followed by body donation is rare in younger and happens mainly in subjects in bad shape. Additionally, the elders may have modified white matter microstructure and vascular environment, but their age is representative of the population suffering from a stroke.

Here we described and demonstrated the feasibility of a standardized protocol for brain management, injection material preparation, simultaneous arterial injection, specimen dissection, and image acquisition. This method is a prerequisite for a precise anatomical description of the brain arterial anatomy, specifically of the detailed vascularization of the main association tracts. The technique is also flexible and, due to the gelatin properties, the prepared specimens can be dissected, serially sectioned for histology, or MR-scanned. The data acquired would provide essential information to better understand the clinical consequences of vascular occlusion by decoding the disconnection syndromes it induces. In addition, precise information on the brain vasculature is crucial for validation of various imaging techniques^59^ and its inclusion in the advanced models used to decode the brain microarchitecture^52^.

## Data Availability

Raw data were generated at Inserm U1253 “Imaging and Brain”, Tours, France. Derived data supporting the findings of this study are available from the corresponding author on request.
